# Selected indicators and determinants of women’s health in the vicinity of a copper mine development in northwestern Zambia

**DOI:** 10.1186/s12905-018-0547-7

**Published:** 2018-05-01

**Authors:** Astrid M. Knoblauch, Mark J. Divall, Milka Owuor, Gertrude Musunka, Anna Pascall, Kennedy Nduna, Harrison Ng’uni, Jürg Utzinger, Mirko S. Winkler

**Affiliations:** 1 Swiss Tropical and Public Health Institute, Basel, Switzerland; 2 University of Basel, Basel, Switzerland; 3 SHAPE Consulting Ltd, St Peter Port, Guernsey, Channel Islands, Guernsey; 4 First Quantum Minerals Limited, Lusaka, Zambia; 5 Solwezi District Health Management Team, Solwezi, Zambia

**Keywords:** Health impact assessment, HIV/AIDS, Knowledge, attitudes and practices (KAP), Women’s health, Maternal health, Migration, Resource extraction, Mining, Resettlement, Zambia

## Abstract

**Background:**

Large projects in the extractive industry sector can affect people’s health and wellbeing. In low- and middle-income countries (LMICs), women’s health is of particular concern in such contexts due to potential educational and economic disadvantages, vulnerability to transactional sex and unsafe sex practices. At the same time, community health interventions and development initiatives present opportunities for women's and maternal health.

**Methods:**

Within the frame of the health impact assessment (HIA) of the Trident copper mining project in Zambia, two health surveys were conducted (baseline in 2011 and follow-up in 2015) in order to monitor health and health-related indicators. Emphasis was placed on women residing in the mining area and, for comparison, in settings not impacted by the project.

**Results:**

All measured indicators improved over time, regardless of whether communities were affected by the project or not. Additionally, the percentage of mothers giving birth in a health facility, the percentage of women who acknowledge that HIV cannot be transmitted by witchcraft or other supernatural means and the percentage of women having ever tested for HIV showed a significant increase in the impacted sites but not in the comparison communities. In 2015, better health, behavioural and knowledge outcomes in women were associated with employment by the project (or a sub-contractor thereof), migration background, increased wealth and higher educational attainment.

**Conclusions:**

Our study reveals that natural resource development projects can positively impact women’s health, particularly if health risks are adequately anticipated and managed. Hence, the conduct of a comprehensive HIA should be a requirement at the feasibility stage of any large infrastructure project, particularly in LMICs. Continued monitoring of health outcomes and wider determinants of health after the initial assessment is crucial to judge the project’s influence on health and for reducing inequalities over time.

**Electronic supplementary material:**

The online version of this article (10.1186/s12905-018-0547-7) contains supplementary material, which is available to authorized users.

## Background

The Sustainable Development Goal (SDG) 2030 agenda situates health as a central feature of all three dimensions of sustainable development: society, economy and environment. It recognises that health is an important contributor to, and beneficiary of, development [1, 2]. The SDGs specifically point at the need for multisectoral collaboration and shared responsibilities in order to safeguard human health through participation, accountability and information sharing mechanisms to ultimately promote synergies between sectors [3]. Therefore, potential health risks due to sectors other than health, including the extractive industry (e.g. oil, gas and mining) should be identified at the feasibility stage and subsequently be mitigated and monitored by means of health-related targets, including social, economic and environmental determinants of health [1, 4].

In 2008, the planning of the Trident project, a greenfields copper development operated by First Quantum Minerals Limited (FQML), commenced in the Northwestern province of Zambia [5]. The project is located in a remote, rural area with a poor health system, and hence, the local population is vulnerable to ill-health [6]. As other natural resource development projects in sub-Saharan Africa, the Trident project potentially influences on all three dimensions of sustainable development: society (e.g. through in-migration and disruption of social cohesion) [7, 8]; economy (e.g. through shifts in occupational activities, increased disposable income or potential inflation of food or goods prices) [9]; and environment (e.g. through the alteration of ecosystems, mining and associated infrastructures) [10, 11].

In sub-Saharan Africa, countries still lack regulatory mechanisms that require large infrastructure development projects to comprehensively assess and manage potential community health impacts. Multilateral lender performance standards are an important platform for promoting health impact assessment (HIA) as part of feasibility studies [12–14]. The financing institutions supporting the Trident project requested that an HIA be conducted, including the systematic identification and management of potential health impacts [15]. To support the monitoring of trends in health outcomes and associated factors throughout the project lifecycle, the community health management plan that resulted from the HIA recommended continuous and periodic data collection, including district health information system data and repeated cross-sectional health surveys at a 4-year interval. Accordingly, the project conducted a baseline health survey (BHS) in July 2011 and a follow-up health survey 4 years later in July 2015. Both surveys included a broad spectrum of biomedical, environmental, structural, behavioural and knowledge indicators in children and adults residing in the project area, as well as in communities outside the project’s area of influence for comparison. In the 2015 survey, it was found that children considered impacted by the project (e.g. in-migrant or resettled children) showed better health (e.g. lower prevalence of stunting) [16]. Although the cross-sectional nature of the study does not allow inferring causality, the results might suggest that the development of large infrastructure projects holds promise to improve children’s health.

Women’s and maternal health is equally important, also when considering that women are often the gatekeepers of family and children in particular. Women and mothers might be affected by such project developments as educational and economic disadvantages, vulnerability to transactional sex and unsafe sex practices, disruption of social cohesion and mental stress render them particularly vulnerable [17, 18]. On the other hand, health interventions and community development initiatives (e.g. women empowerment and targeted educational programmes) initiated or supported by extractive industry projects offer opportunities to improve their health [11, 19].

Here, we present a selection of health outcomes and behavioural and knowledge indicators in women of reproductive age. While a broad spectrum of indicators was assessed, data presented here have a focus on women’s health. Table 1 summarises the selected indicators and highlights their relevance in the given context. We compare results from the 2011 BHS with the 2015 follow-up survey and analyse associated socioeconomic, structural and setting-specific factors such as employment, migration and resettlement. Findings are discussed in the context of HIA applied in infrastructure development projects as an approach to foster health equity and sustainable development.

**Table 1:** Selected indicators in women of reproductive age and their relevance to women’s health in the Trident copper mining project area, Northwestern province, Zambia

Indicator	Definition/description	Relevance to women’s health and the local project context
Percentage of anaemia in women of reproductive age	Pregnant women: haemoglobin (Hb) < 11 g/dl; non-pregnant women: Hb < 12 g/dl [40].	Anaemia is considered as a proxy indicator for general health and wellbeing. Anaemia has been associated with reduced work capacity, fatigue, reduced ability to execute routine daily activities, reduced cognitive function, poor pregnancy outcomes and negative effects on foetal and child health [41, 42]. Epidemiology of infectious diseases, access to health care and nutrition potentially change due to the project development which, in turn, influence rates of anaemia [26].
Percentage of women with past and current syphilis infection	Antibodies to *Treponema pallidum* assessing past or current syphilis infection [38].	Syphilis renders women more susceptible to an HIV infection and increases viral loads in HIV-infected individuals [24]. Untreated syphilis causes perinatal deaths and congenital syphilis in children, which impairs child health [43]. High increases of sexually transmitted infections (STIs) other than HIV have been reported in mining areas [44].
Percentage of women who delivered their last born child at a health facility	Percentage of women with a child under 5 years of age who delivered their last born child at a public or a private health facility [40].	Increasing the percentage of births delivered in health facilities is an important factor in reducing deaths arising from complications of pregnancy provided a skilled attendant can manage complications during delivery or refer the mother to the next level of care in a timely manner [40]. The Trident project can influence rates of deliveries at health facilities through increased access because of improved roads or increased financial means to pay for maternal health services.
Percentage of women with comprehensive knowledge on HIV/AIDS	Comprehensive knowledge means knowing that consistent use of a condom during sexual intercourse and having just one uninfected, faithful partner can reduce the chance of getting HIV, knowing that a healthy-looking person can have HIV, and rejecting the two most common local misconceptions, i.e. HIV can be transmitted by mosquito bites or supernatural means [40].	Correct knowledge can influence an individual’s ability to adopt safer sex practices, reduce stigmatisation towards people living with HIV/AIDS and alleviate misconceptions related to HIV/AIDS [40]. The increased spread of HIV/AIDS characteristic to mining areas has been widely described [40, 45]. In the Trident project HIA, the transmission of STIs, including HIV/AIDS, was identified as priority and an HIV/AIDS intervention package was implemented early on in the project development in the workforce and the communities [22] (see also health road shows below).
Percentage of women who believe HIV can be transmitted by witchcraft or supernatural means	Belief that HIV can be transmitted by witchcraft or supernatural means [40].	Knowledge of HIV status is important for helping individuals decide to adopt safer sex practices and to reduce their risk of becoming infected or transmitting HIV [40]. HIV testing and counselling is one of the major health interventions supported by FQML in the frame of the health road shows.
Percentage of women who ever tested for HIV	Percentage of women who ever tested for HIV [40].	
Percentage of women who participated in a health road show	A health road show is a 1-day visit to communities in the project area involving information, education and communication (IEC) and biomedical testing for HIV, glycaemia, blood pressure and malaria [22].	Health road shows are one of the major health interventions initiated by FQML. While uptake of biomedical testing offered at a health road show is recorded, the influence of the IEC campaigns has yet to be determined.

## Methods

### Ethical considerations

The two cross-sectional health surveys conducted in 2011 and 2015 were approved by the ethics review committee of the Tropical Disease Research Centre (Ndola, Zambia; registration number 00003729). Written informed consent was obtained from participating women (fingerprint for illiterate subjects). Study participants found anaemic were started on haematinics, while severe cases were referred to the health system. Women found positive for syphilis were given treatment on site together with their sexual partner(s), using a single dose of 2 g of azithromycin.

### Study area

**Fig. 1:**
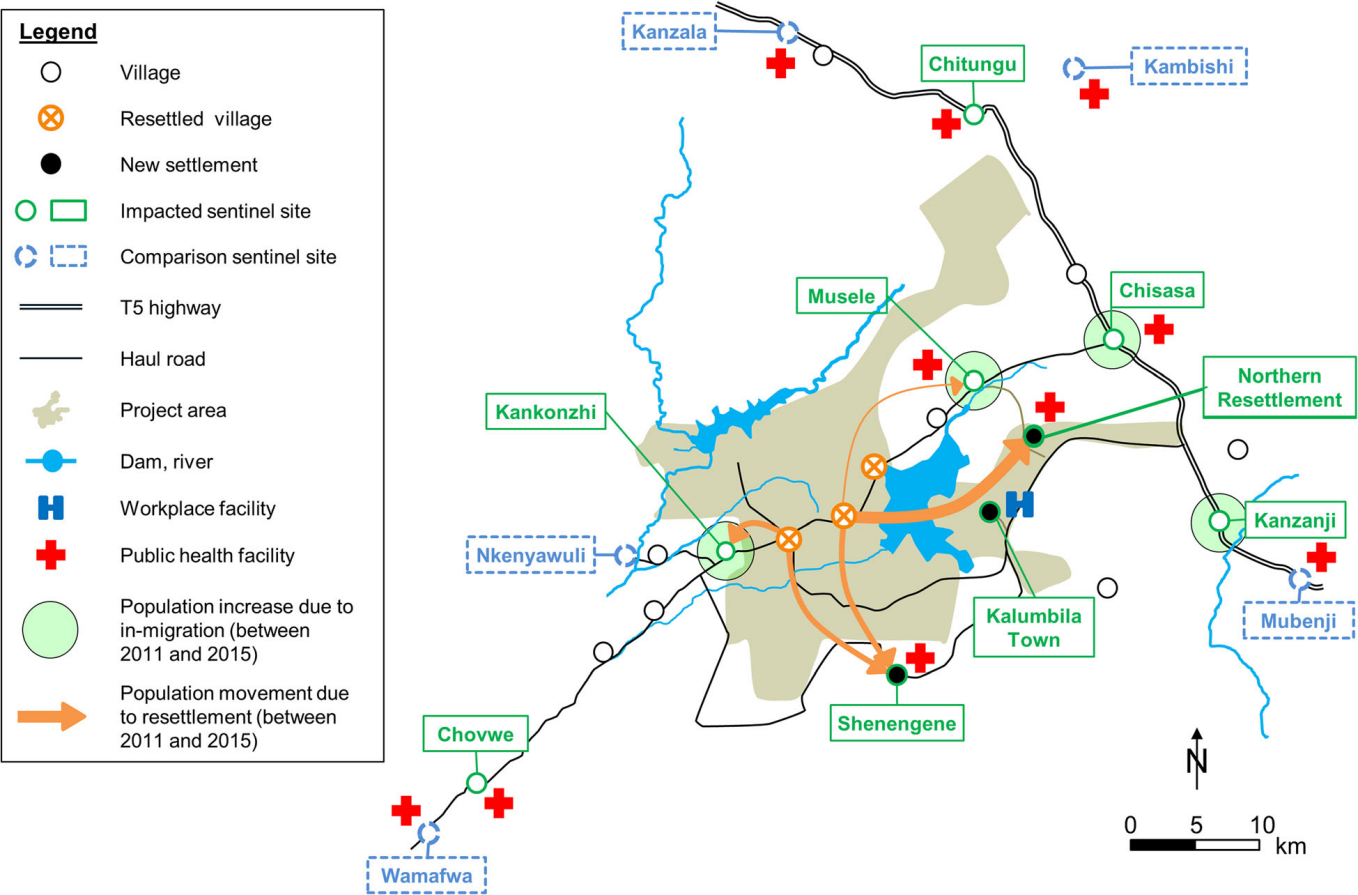
Study area, demographic developments between 2011 and 2015 and selected sentinel sites in the Trident copper mining project area, Northwestern province, Zambia

The Trident copper mining project is located in the Musele chiefdom, Solwezi district, Northwestern province of Zambia. The project concession area covers 950 km^2^, which accommodates the open pit mine, project infrastructure (e.g. roads, processing plant, airstrip and offices), two newly built dams and a nature and game conservation area. Due to project developments, some villages were resettled, new settlements established, new roads constructed and existing ones upgraded. In-migration resulted in considerable population increases and rapid urbanisation. Figure 1 shows the project area and surroundings, including demographic changes that occurred between the BHS in 2011 and the follow-up survey in 2015.

### Sampling method

A detailed description of the sampling strategy is provided elsewhere [20]. In brief, the study pursued a cross-sectional design with a baseline survey in 2011 and a follow-up survey in 2015. A sentinel site approach was chosen in order to be able to purposively select specific settlements that are impacted by the project but might have been missed in a probability sampling. Comparison sites were selected based on (i) proximity to the project area; (ii) similarity to impacted sentinel sites; and (iii) minimal predicted impact due to the project. Within the sentinel sites, households were randomly selected using a quota sampling of at least 25 households per sentinel site. The presence of a woman with a young child (< 5 years of age) served as the key inclusion criterion.

### Data collection

A questionnaire was administered for eligible women to obtain socio-demographic background information, health-related knowledge, attitudes and practices (KAP), information on maternal health, exposure to health interventions and migration background (migration was defined as duration of residency in the current location of less than 5 years). The questionnaire is provided in Additional file 1. Following the questionnaire interview, women were invited to visit a mobile field laboratory located within the sentinel site to have several biomedical indicators measured. First, a HemoCue® 201+ testing device (HemoCue Hb 201 System; HemoCue AB, Ängelholm, Sweden) was employed to determine haemoglobin (Hb) concentration in a capillary blood sample as a proxy for anaemia. Second, detection of antibodies due to *Treponema pallidum* was done using Alere Determine™ Syphilis TP rapid diagnostic test (RDT) to assess for current and past syphilis infection (Alere Determine™ Syphilis; Alere Inc., Waltham, MA, USA).

### Statistical analysis

In 2011, data were collected with a paper-based questionnaire and entered into EpiData version 1.4.4 (EpiData Association; Odense, Denmark). In 2015, data were collected electronically, with tablets using Open Data Kit (ODK) software. Analysis was performed with Stata version 13 (StataCorp LP; College Station, TX, USA). Principal component analysis (PCA) based on 18 household assets was used to approximate household wealth, with households stratified into four wealth quartiles [21].

## Results

### Study population

There were seven sentinel sites in the BHS in 2011. Four years later, an additional three impacted sites were included to represent newly developed settlements or settlements that gained importance due to project implementation. Four comparison sentinel sites were also included, and hence, in 2015, a total of 14 sentinel sites were included. Table 2 shows the study population in the two cross-sectional surveys, stratified by sentinel site. In 2011, 286 women of childbearing age were sampled. In the follow-up survey in 2015, there were 606 women.

**Table 2:** Study populations in the two cross-sectional surveys done in 2011 and 2015 and community characteristics in 2015, Trident copper mine project, Northwestern province, Zambia

Sentinel site	Women aged 15–49 years		% of HH that have been resettled due to the project	% of migrant HH (in the area for < 5 years)	% of HH with at least one HH member being an employee of FQML or a contractor	% of HH in the richest wealth quartile
Year	2011	2015	2015	2015	2015	2015
Kalumbila Town	n/a	31	0.0	100.0	79.3	100.0
Wanyinwa (2011) / Northern Resettlement (2015)	35	41	96.9	3.1	75.0	56.3
Shenengene	n/a	35	96.9	3.1	40.6	28.1
Musele^a^	30	87	3.2	30.2	38.1	30.2
Chisasa^a^	63	75	1.6	65.1	39.7	31.8
Kankonzhi^a^	36	38	3.5	37.9	75.9	31.0
Chovwe^a^	61	38	0.0	6.3	9.4	6.3
Kanzanji	n/a	38	3.1	43.8	28.1	21.9
Chitungu^a^	30	38	0.0	0.0	0.0	3.0
Total impacted	255	421	19.4	34.2	41.6	33.0
Nkenyawuli^a^	31	31	0.0	6.9	3.5	6.9
Wamafwa	n/a	38	0.0	6.3	0.0	6.3
Kanzala	n/a	35	0.0	16.7	0.0	3.3
Kambishi	n/a	38	0.0	0.0	0.0	3.1
Mubenji	n/a	43	0.0	21.2	24.2	27.3
Total comparison	31	185	0.0	26.8	5.8	9.6

FQML, First Quantum Minerals Limited; HH, household; n/a, not available

^a^Sentinel site with data for 2011 and 2015

### HIV-related indicators

**Fig. 2:**
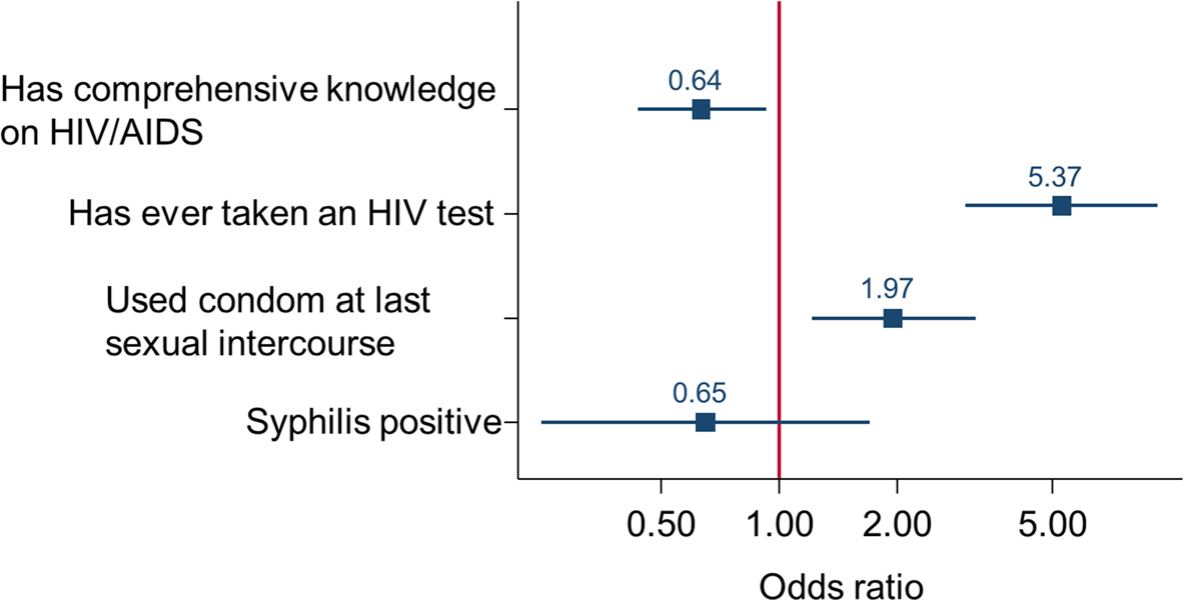
Knowledge and behavioural outcomes in women of reproductive age who participated in health road shows in 2011 and 2015, Trident copper mining project, Northwestern Province, Zambia

Comprehensive knowledge pertaining to HIV/AIDS in women of reproductive age increased non-significantly from 26.2% (95% confidence interval (CI) 21.2–31.7%) to 34.1% (95% CI 30.3–38.0%) (Table 3). In 2015, women who attained secondary schooling or higher and women belonging to the richest wealth quartile were found with significantly higher levels of comprehensive knowledge on HIV/AIDS than their less educated or poorest counterparts. In 2015, 15.1% (95% CI 12.4–18.2%) of women of reproductive age still believed that HIV can be transmitted by witchcraft or other supernatural means, which was, however, almost half the percentage reported in 2011 (26.2%, 95% CI 21.2–31.7%). Participation in ‘health road shows’ (recurring 1-day visits to communities in the project area, involving information, education and communication (IEC) on, and testing for, HIV [22]) was not associated with better knowledge on HIV/AIDS. As seen in Fig. 2, the odds of having comprehensive knowledge on HIV/AIDS was significantly lower in women who participated in health road shows (odds ratio (OR) = 0.64, 95% CI 0.44–0.93).

**Table 3:** Knowledge and behavioural indicators related to HIV/AIDS in women of reproductive age, 2011 and 2015, Trident copper mining project, Northwestern province, Zambia

Indicator	n		% of women with comprehensive knowledge on HIV/AIDS^1^		% of women who believe HIV can be transmitted by witchcraft or supernatural means		% of women who have ever tested for HIV	
	2011	2015	2011	2015	2011	2015	2011	2015
Health road show								
Did not participate	n/a	287	n/a	37.2 (31.6–43.1)	n/a	12.1 (8.6–16.5)	n/a	74.9 (69.4–79.8)
Participated	n/a	319	n/a	31.3 (26.2–36.7)	n/a	17.8 (13.8–22.5)	n/a	94.0 (90.8–96.3) ^†^
Education								
No education	100	345	25.0 (16.8–34.6)	26.6 (22.0–31.6)	33 (23.9–43.1)	16.2 (12.5–20.5) ^ɸ^	71 (61.0–79.6)	81.4 (76.9–85.4)
Primary school	186	207	26.8 (20.6–33.8)	37.6 (31.0–44.6)	22.5 (16.7–29.2)	13.5 (9.2–18.9)	77.4 (70.7–83.2)	88.4 (83.2–92.4)
Secondary or higher	0	54	n/a	68.5 (54.4–80.4) ^†^	n/a	14.8 (6.6–27.1)	n/a	94.4 (84.6–98.8)
FQML or contractor employment within the HH								
No	n/a	422	n/a	32.4 (28.0–37.1)	n/a	13.2 (10.1–16.8)	n/a	80.8 (76.7–84.4)
Yes	n/a	184	n/a	38.0 (31.0–45.4)	n/a	19.5 (14.0–26.0)	n/a	94.5 (90.2–97.3) ^†^
Resettlement								
No	n/a	527	n/a	33.3 (29.3–37.6)	n/a	15.5 (12.5–18.9)	n/a	83.4 (80.0–86.5)
Yes	n/a	79	n/a	39.2 (28.4–50.8)	n/a	12.6 (6.2–22.0)	n/a	94.9 (87.5–98.6) ^†^
Migration background								
No	n/a	447	n/a	31.0 (26.8–35.6)	n/a	14.9 (11.8–18.6)	n/a	82.5 (78.7–85.9)
Yes	n/a	159	n/a	42.7 (34.9–50.8)	n/a	15.7 (10.4–22.3)	n/a	91.8 (86.4–95.5) ^†^
Asset-based wealth index								
Poorest	n/a	167	n/a	26.3 (19.8–33.7)	n/a	17.9 (12.4–24.6)	n/a	74.8 (67.5–81.2)
Second	n/a	132	n/a	27.2 (19.8–35.7)	n/a	15.9 (10.1–23.2)	n/a	85.6 (78.4–91.1)
Third	n/a	152	n/a	33.5 (26.1–41.6)	n/a	11.8 (7.2–18.0)	n/a	87.5 (81.1–92.3)
Richest	n/a	155	n/a	49.0 (40.9–57.1) ^†^	n/a	14.8 (9.6–21.4)	n/a	92.9 (87.6–96.4) ^†^
Health facility within the community								
No	67	107	32.8 (21.8–45.3)	26.1 (18.1–35.5)	19.4 (10.7–30.8)	21.4 (14.1–30.4)	77.6 (65.7–86.8)	79.4 (70.5–86.6)
Yes	219	499	24.2 (18.6–30.4)	35.8 (31.6–40.2) ^ɸ^	28.3 (22.4–34.7)	13.8 (10.9–17.1) ^ɸ^	74.4 (68.1–80.0)	86.3 (83.0–89.2) ^ɸ^
Impact								
Impacted	255	421	26.2 (20.9–32.1)	32.7 (28.3–37.4)	26.6 (21.3–32.5)	16.6 (13.1–20.5) ^ɸ^	76.0 (70.3–81.1)	90.0 (86.7–92.7) ^ɸ^
Comparison	31	185	25.8 (11.8–44.6)	37.2 (30.3–44.6)	22.5 (9.59–41.0)	11.8 (7.6–17.4)	67.7 (48.6–83.3)	73.5 (66.5–79.7) ^†^
Total	286	606	26.2 (21.2–31.7)	34.1 (30.3–38.0)	26.2 (21.2–31.7)	15.1 (12.4–18.2) ^ɸ^	75.1 (69.7–80.0)	84.9 (81.8–87.7) ^ɸ^

FQML, First Quantum Minerals Limited; HH, household; n/a, not available

^1^Knowing that consistent use of condoms during sexual intercourse and having just one uninfected faithful partner can reduce the chance of getting HIV, knowing that a healthy-looking person can have HIV, and rejecting the two most common local misconceptions, i.e. HIV can be transmitted by mosquito bites or supernatural means

^ɸ^Significant difference between 2011 and 2015

^†^Significant difference between population sub-groups in 2015

The proportion of women who underwent an HIV test was significantly higher in 2015 (84.9%, 95% CI 81.8–87.7%) compared to the baseline in 2011 (75.1%, 95% CI 69.7–80.0%). This trend towards testing for HIV status was significant in impacted sites (76.0%, 95% CI 70.3–81.1% in 2011 versus 90.0%, 95% CI 86.7–92.7% in 2015) but not in comparison sites (67.7%, 95% CI 48.6–83.3% in 2011 versus 73.5%, 95% CI 66.5–79.7% in 2015). Consequently, the HIV testing coverage in 2015 was significantly higher in the impacted versus the comparison sites. In 2015, the odds of having done an HIV test was 5.37 (95% CI 3.06–9.42) in individuals who participated in a health road show (Fig. 2), compared to women never participating in a health road show. Moreover, having a household member employed by FQML or in some way contracted to the mine, having been resettled, having a migration background or belonging to the richest wealth quartile, has significantly increased testing rates in women of reproductive age.

### Place of delivery of the last-born child

The proportion of women who gave birth to their last-born child at a health facility was significantly higher in 2015 (80.3%, 95% CI 76.9–83.4%) compared to the baseline in 2011 (65.0%, 95% CI 59.1–70.5%; Table 4). This trend was significant in the impacted sites (83.3%, 95% CI 79.4–86.8% in 2015 compared to 66.6%, 95% CI 60.5–72.4% in 2011) and in communities that had a health facility within the settlement (81.1%, 95% CI 77.4–84.5% in 2015 versus 66.2%, 95% CI 59.5–72.4% in 2011). In 2015, women who attained at least secondary schooling, those with a migration background and those belonging to the richest quartile were significantly more likely to deliver at a health facility than the other sub-groups.

**Table 4:** Behavioural and health outcome indicators in women of reproductive age, 2011 and 2015, Trident copper mining project, Northwestern province, Zambia

Indicator	n*		Delivery at a health facility (%; 95% CI)		Anaemia (%; 95% CI)		Syphilis in women aged 15–49 years (%; 95% CI)*	
	2011	2015	2011	2015	2011	2015	2011	2015
Education								
No education	100	345	49.0 (38.8–59.1)	75.3 (70.4–79.8) ^ɸ^	19.6 (12.7–28.2)	27.5 (22.8–32.5)	n/a	4.9 (2.8–7.9)
Primary school	186	207	73.6 (66.7–79.8)	85.0 (79.4–89.5)	17.1 (12.2–23.0)	24.1 (18.4–30.5)	n/a	1.6 (0.3–4.7)
Secondary or higher	0	54	n/a	94.4 (84.6–98.8) ^†^	n/a	16.6 (7.9–29.2)	n/a	6.0 (1.3–16.5)
FQML or contractor employment within the HH								
No	n/a	422	n/a	77.9 (73.6–81.8)	n/a	27.4 (23.2–32.0)	n/a	4.4 (2.6–7.0)
Yes	n/a	184	n/a	85.8 (79.9–90.5)	n/a	20.6 (15.0–27.2)	n/a	2.9 (0.9–6.5)
Resettlement								
No	n/a	527	n/a	81.5 (78.0–84.8)	n/a	25.9 (22.3–29.9)	n/a	3.7 (2.2–5.8)
Yes	n/a	79	n/a	72.1 (60.9–81.6)	n/a	21.5 (13.0–32.2)	n/a	5.3 (1.5–13.0)
Migration background								
No	n/a	447	n/a	76.7 (72.5–80.5)	n/a	25.5 (21.5–29.8)	n/a	4.2 (2.4–6.6)
Yes	n/a	159	n/a	90.5 (84.9–94.6) ^†^	n/a	25.1 (18.6–32.6)	n/a	3.3 (1.1–7.6)
Asset-based wealth index								
Poorest	n/a	167	n/a	71.8 (64.3–78.5)	n/a	28.7 (22.0–36.2)	n/a	5.4 (2.4–10.3)
Second	n/a	132	n/a	78.0 (69.9–84.7)	n/a	21.2 (14.5–29.1)	n/a	4.8 (1.8–10.1)
Third	n/a	152	n/a	84.8 (78.1–90.1)	n/a	26.3 (19.5–34.0)	n/a	2.9 (0.8–7.2)
Richest	n/a	155	n/a	87.0 (80.7–91.9) ^†^	n/a	24.5 (17.9–32.0)	n/a	2.7 (0.7–6.8)
Health facility within the community								
No	67	107	61.1 (48.5–72.8)	76.6 (67.4–84.2)	14.2 (7.4–24.1)	27.1 (18.9–36.5)	n/a	6.7 (2.7–13.2)
Yes	219	499	66.2 (59.5–72.4)	81.1 (77.4–84.5) ^ɸ^	19.2 (14.4–24.8)	25.0 (21.3–29.0)	n/a	3.3 (1.9–5.4)
Impact								
Impacted	255	421	66.6 (60.5–72.4)	83.3 (79.4–86.8) ^ɸ^	17.1 (12.9–22.0)	25.1 (21.1–29.6)	n/a	4.3 (2.5–6.8)
Comparison	31	185	51.6 (33.0–69.8)	73.5 (66.5–79.7)	25.8 (11.8–44.6)	25.9 (19.7–32.8)	n/a	3.1 (1.0–7.0)
Total	286	606	65.0 (59.1–70.5)	80.3 (76.9–83.4) ^ɸ^	21.2 (16.8–26.1)	25.4 (21.9–29.0)	n/a	3.9 (2.5–5.9)

FQML, First Quantum Minerals Limited; HH, household; n/a, not available

*Denominators differ for syphilis as not all women were tested

^ɸ^Significant difference between 2011 and 2015

^†^Significant difference between population sub-groups in 2015

### Health outcomes: anaemia and syphilis

As shown in Table 4, among all women subjected to Hb measurements, 21.2% (95% CI 16.8–26.1%) in 2011 and 25.4% (95% CI 21.9–29.0%) in 2015 were found anaemic. The slightly higher proportion of anaemia in the 2015 follow-up survey lacked statistical significance. No differences were found between sub-groups stratified for different background characteristics. Women with secondary education or higher were least affected by anaemia (16.6%, 95% CI 7.9–29.2%).

The overall syphilis prevalence in women of reproductive age in 2015 was 3.9% (95% CI 2.5–5.9%), whilst no significant differences were found when stratified by background characteristics. Women residing in the impacted sites had a higher prevalence (4.3%, 95% CI 2.5–6.8%), compared to women in the comparison sites (3.1%, 95% CI 1.0–7.0%) although the observed difference lacked statistical significance. Women who participated in the health road shows were at a somewhat lower odds of syphilis infection, yet without a statistically significant difference (OR = 0.65, 95% CI 0.25–1.73; Fig. 2).

## Discussion

Women of reproductive age residing in a newly developed copper mining area in northwestern Zambia were subjected to a number of health and KAP indicators in two cross-sectional surveys; a baseline in 2011 and a follow-up survey 4 years later. Several indicators improved over time in the impacted sentinel sites but not in the comparison sites: (i) the percentage of mothers giving birth in a health facility; (ii) the percentage of women who acknowledge that HIV cannot be transmitted by witchcraft or other supernatural means; and (iii) the percentage of women having ever tested for HIV. In 2015, employment in the project or as a contractor, migration background, belonging to the richest wealth quartile and educational attainment of at least secondary schooling were associated with better health and behavioural and knowledge outcomes in women reflecting a social gradient in health [23].

The health road show is a major project-initiated intervention, primarily focussed on the prevention of sexually transmitted infections (STIs). Interestingly, it failed to translate into better knowledge on HIV/AIDS in women of reproductive age. Indeed, women who did not participate in these activities had higher levels of comprehensive knowledge on HIV/AIDS and lower levels of believing that HIV can be transmitted by supernatural means. Although women with a migrant background had higher levels of comprehensive knowledge overall (42.8%, 95% CI 35.0–50.8%) – probably acquired elsewhere before in-migration – than women without a migrant background (31.1%, 95% CI 26.8–35.6%), this difference was not significant. Importantly though, the promotion activities had a positive effect on HIV-testing uptake and condom use, as determined by the most recent sexual intercourse, both key prevention methods against STIs. In fact, HIV-testing uptake has increased markedly in the study area since the initiation of the health road shows and the HIV positivity rate observed through continuous HIV testing and counselling offered in the communities surrounding the project was similar in 2012 (3.0%, 95% CI 2.0–4.2%) and 2015 (3.4%, 95% CI 2.9–3.9%) [22]. The interplay of HIV with other STIs ought to be considered in this setting. For instance, syphilis renders women more susceptible to an HIV infection and increases viral loads in HIV-infected individuals, and thus individuals become more infectious [24]. Syphilis infection in the project area was similar to what was found in the 2007 Demographic and Health Survey (DHS) in Zambia. The prevalence was 3.3% in women in Northwestern province and 3.9% in women living in rural areas, compared to a prevalence of 3.9% in our 2015 follow-up survey [25].

The stable anaemia rate over the 4-year period and the low variation among the different population strata underlines the slow progress made to improve anaemia in women of reproductive age, perhaps due to persistent micro-nutrient deficiencies, inherited disorders and sustained parasitic infections (e.g. hookworm and malaria) in the present setting [26]. As reported elsewhere, the malaria prevalence assessed during the same surveys in children under 5 years of age has almost doubled. Hence, it is conceivable that malaria in women, including pregnant women, has also increased [16]. A quarter of women belonging to the richest quartile was anaemic, although they presumably have sufficient energy intake and lower rates of infectious diseases compared to their poorer counterparts [27]. A deeper assessment of nutritional patterns in women of reproductive age will reveal why iron needs are not met [28].

Taken together, the results from women presented here and from children communicated elsewhere [16] suggest that the in-migrating population is generally healthier than the local host population. The ‘healthy migrant hypothesis’ speculates that although migrants face disadvantages in a new environment, which can affect their health, they are a selectively healthier group compared to non-migrants [29]. This hypothesis cannot be tested in the current setting as pre-migration data from migrants are not available but the characteristics of the migrants in this area, i.e. young labour-seekers, suggest that they are in good physical health and the current data might support the ‘healthy migrant hypothesis’. At the same time, this underlines the particular attention that should be paid to the native population which are predisposed to economic and social vulnerability, including health, and have a low assimilative capacity to changes brought about by immigrants [8].

Another finding further raises concern related to equity; women from households where one or more persons are employed or contracted by the project showed systematically better outcomes for the indicators assessed, compared to their counterparts. Economic growth, brought by activities such as mining, does not automatically translate into an equal distribution of benefits but needs social policies to create a fair environment, including health equity [30, 31]. Effectively, there are socioeconomic or environmental domains that are outside the influence of project decision-makers and the local government has a role in supporting an equal opportunity environment [32]. Furthermore, the HIA’s claim to reduce inequalities has yet to be validated [33, 34]. Repeated cross-sectional monitoring over a project’s lifespan will provide valuable information on HIA’s performance if applied in infrastructure development projects in remote rural Africa. Shortcomings of the cross-sectional design are, however, that it is (i) unlikely to detect rare diseases; (ii) biased towards chronic conditions; and (iii) unable to establish the sequence of events (association versus causality) [35]. Hence, to validate the impact assessment’s promise to support sustainable development around infrastructure development projects, continued long-term environmental, social and health surveillance alongside cross-sectional monitoring is indispensable [11, 36]. This is ideally achieved through health systems strengthening or even prospective cohort studies within HIA.

Thus far, the HIA for the Trident copper mining project has demonstrated that (i) the community health management plan was tailored based on local health needs; (ii) close collaboration between the private and the health sector was achieved as the project acts as an implementing partner to the district health management team; and (iii) baseline and follow-up surveys are crucial for benchmarking and monitoring of the health status of communities residing in the project area and to evaluate the performance of health interventions. Importantly, given the systematic and comprehensive approach to health, which includes wider determinants of health, HIA aligns with the health-in-all sectors approach proposed in the SDGs [1]. However, it is too premature to judge the project’s overall influence on sustainable development, which is one of HIA’s core values and a declared goal of FQML’s operations [15, 37].

### Limitations

The Alere DetermineTM Syphilis TP detects antibodies of all isotypes against *T. pallidum*, the syphilis causing bacteria, and is unable to differentiate between active and cured disease [38]. The prevalence rate reported is therefore a combination of past and current infection. Due to the semi-purposive sentinel site sampling, the results have limited generalisability to other communities. In 2011, a number of indicators (e.g. syphilis, employment or migration background) were not assessed and only one comparison site was sampled, which limited a more comprehensive comparison from baseline to the 2015 follow-up.

## Conclusions

Factors associated with the development of a large copper mine in the Northwestern province of Zambia, such as employment and an individual’s migration background, were associated with better health, behavioural and knowledge outcomes in women of reproductive age residing in the mining area. Although a general improvement of the assessed indicators was observed, the improvements were more articulated in the communities considered impacted by the mine as opposed to comparison communities outside the direct area of influence. Additionally, women with links to project-employment or a migration background showed, on average, better outcomes, indicating inequalities and the need to address the local poor. Nevertheless, the implementation of community health interventions at the Trident project suggests that the development of this copper mine can be seen as an opportunity to improve women’s health if potential health impacts and risk factors are appropriately identified and mitigated. The evidence achieved through cross-sectional monitoring is key in observing the health status of communities impacted by a project. In fact, the HIA process entails a health monitoring component which, however, is currently being under-utilised in natural resource development projects and limits the evaluation on the contributions of such projects to protecting human health and fostering sustainable development [14, 39].

## Additional file

Additional file 1: Survey questionnaire. (XLSX 122 kb)

## Abbreviations

AIDS: Acquired immune deficiency syndrome
BHS: Baseline health survey
CI: Confidence interval
DHS: Demographic and Health Survey
FQML: First Quantum Minerals Limited
Hb: Haemoglobin
HIA: Health impact assessment
HIV: Human immunodeficiency virus
IEC: Information, education and communication
KAP: Knowledge, attitudes and practices
ODK: Open data kit
OR: Odds ratio
PCA: Principal component analysis
RDT: Rapid diagnostic test
SDG: Sustainable Development Goal
STI: Sexually transmitted infection
